# A Case of a Dentigerous Cyst in the Maxillary Sinus Treated Preoperatively With Vascular Embolization to Avoid Intraoperative Abnormal Bleeding

**DOI:** 10.7759/cureus.50228

**Published:** 2023-12-09

**Authors:** Takaharu Taketomi, Takao Fukuda, Go Takeshita, Terukazu Sanui

**Affiliations:** 1 Department of Dental and Oral Surgery, St. Mary’s Hospital, Kurume, JPN; 2 Dental and Oral Medical Center, Kurume University School of Medicine, Kurume, JPN; 3 Department of Dental and Oral Surgery, Takagi Hospital, Okawa, JPN; 4 Department of Periodontology, Division of Oral Rehabilitation, Faculty of Dental Science, Kyushu University, Fukuoka, JPN; 5 Department of Radiology, Takagi Hospital, Okawa, JPN; 6 Department of Radiology, Faculty of Medicine, Saga University, Saga, JPN

**Keywords:** computed tomography angiography, bleeding, vascular embolization, maxillary sinus, dentigerous cyst

## Abstract

Bone cysts involving the maxillary sinus are frequently observed, and controlling bleeding from the maxillary or posterior superior alveolar arteries is extremely difficult when the surgical site extends into the palatine fossa or the wing socket behind the maxillary sinus. In this report, we describe a case wherein preoperative endovascular arterial embolization prevented bleeding owing to an unexpected vascular injury that occurred during the removal of a dentigerous cyst from the maxillary sinus. This resulted in a safe operation with less intraoperative bleeding. Although this approach carries the risk of complications, such as paralysis, around the affected area, the likelihood of such complications is low. This approach is useful for performing a safe surgery with minimal blood loss because it avoids the need for emergency hemostasis for major intraoperative hemorrhage.

## Introduction

Cystic lesions commonly develop in the maxilla without any evident symptoms. Enlarged cysts may extend into the maxillary sinus, causing bone resorption of the posterior wall of the maxillary sinus. In such cases, hemostasis is extremely important for a safe and smooth surgery; inadvertent attempts to remove the cyst may result in massive bleeding owing to damage to the posterior superior alveolar artery or maxillary artery. However, it is extremely difficult to stop massive intraoperative bleeding in emergencies, and an approach to the external carotid artery may be required to stop hemorrhage occurring from the posterior wall of the maxillary sinus, where the operative field is narrow. Endovascular arterial embolization in the maxillofacial region has been reported to be useful for hemostasis in end-stage oral cancer, multiple facial fractures, and sclerotherapy of hemangiomas [1-6]. However, there have been no reports of preoperative vascular embolization being performed to prevent hemorrhage when removing a cyst that is in contact with the posterior wall of the maxillary sinus. This is a novel method that is useful for avoiding the risk of hemorrhage.

In this report, we describe a case wherein endovascular arterial embolization before the removal of a contained cyst in the maxillary sinus was effective in preventing intraoperative hemorrhage.

## Case presentation

**Figure 1:**
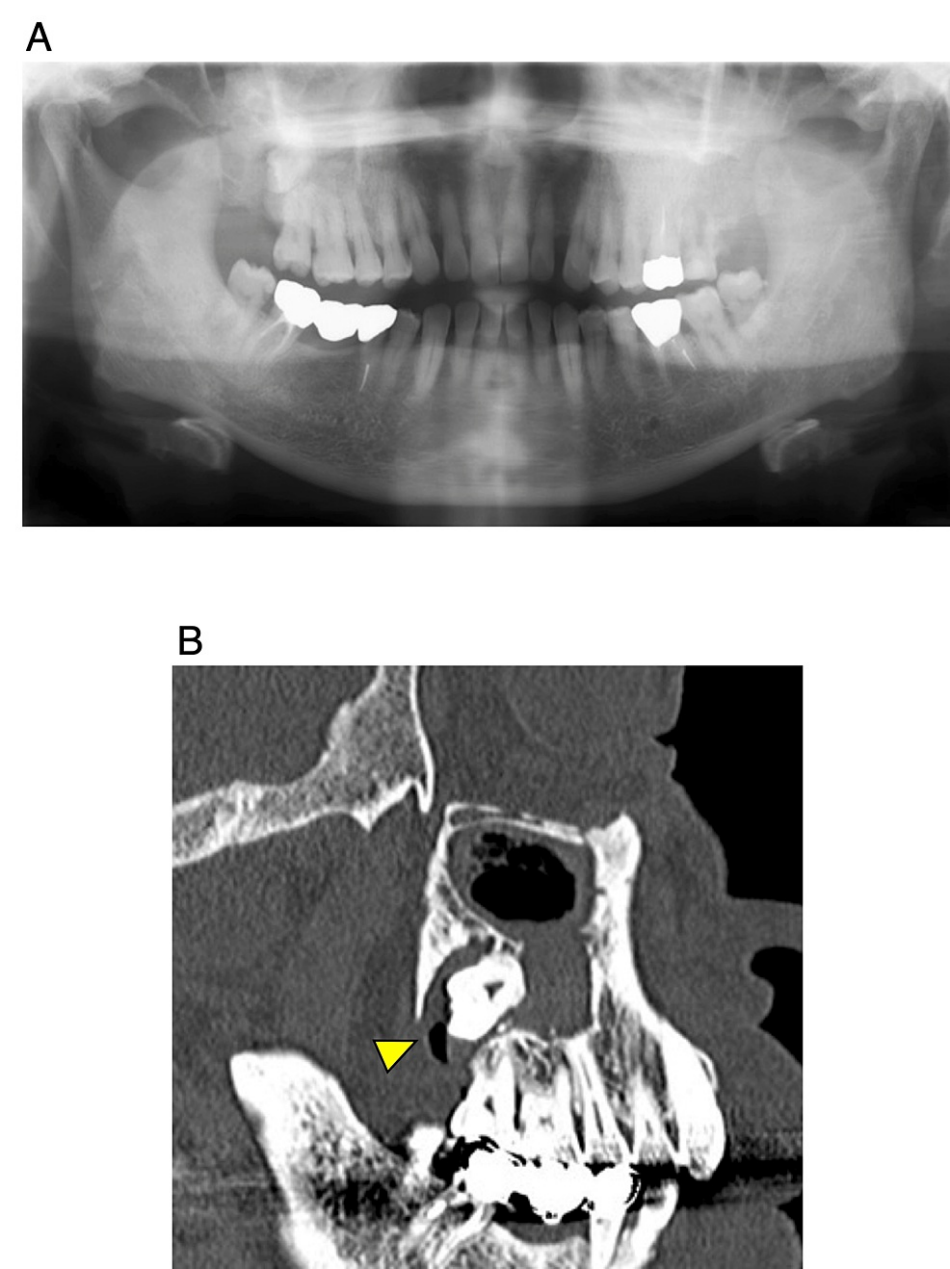
Panoramic radiograph and computed tomography (CT) image. A: Cystic lesion involving the third molar and crown embedded posteriorly in the right maxillary sinus. B: CT image showing the cystic lesion causing absorption of the posterior wall of the maxillary sinus and extending into the pterygopalatine fossa (arrowhead).

A 66-year-old male patient presented to our department with a chief complaint of swelling in the right cheek area. Panoramic radiographs showed a cystic lesion around the third molar and its surrounding area in the right maxillary sinus (Figure 1A); thereafter, computed tomography (CT) was performed (Figure 1B).

**Figure 2:**
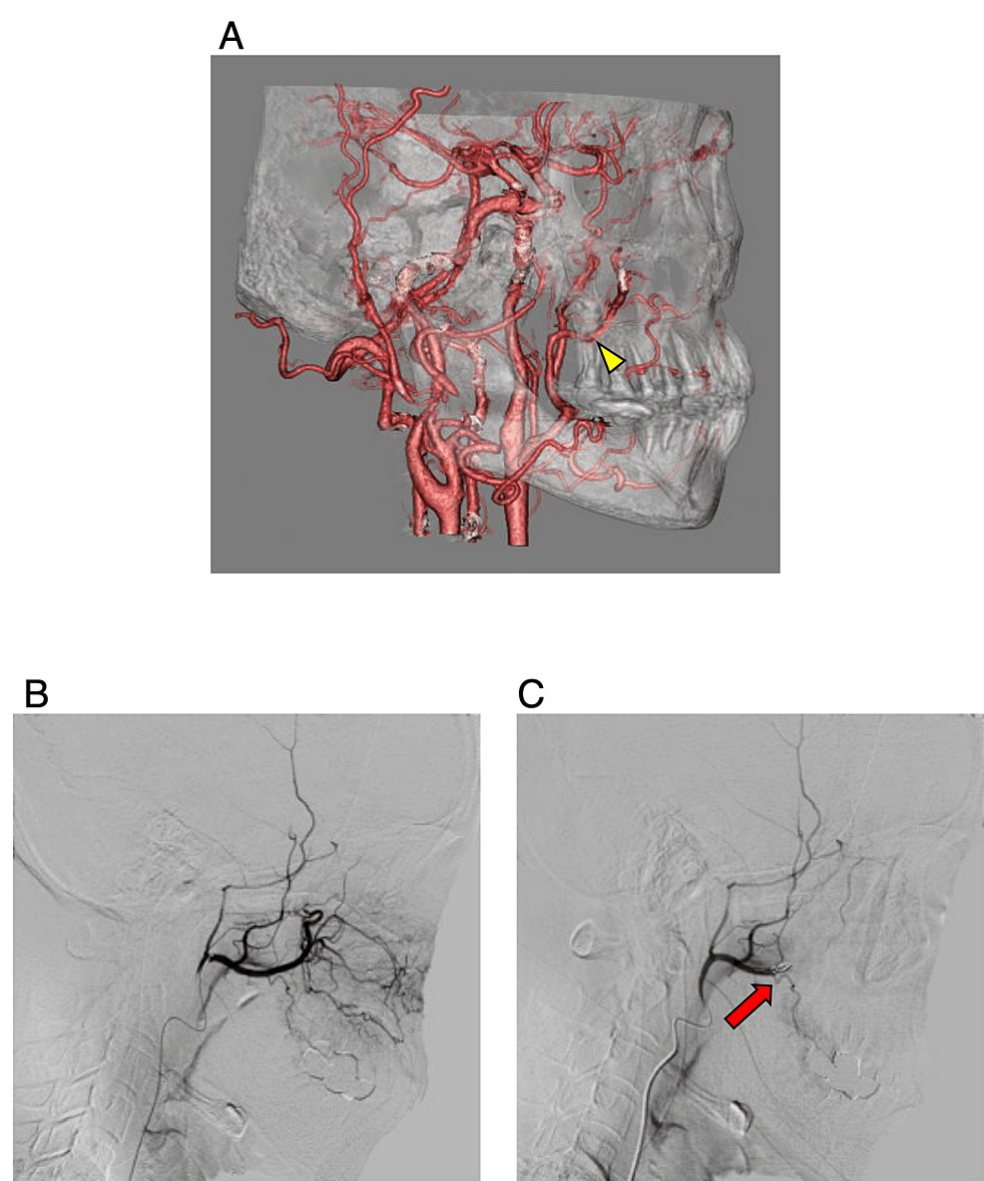
Angiographic images of the maxillary artery and its branches were obtained via CT angiography. A: CT angiography showing a branch of the maxillary and posterior superior alveolar arteries located peripherally from the middle meningeal artery (arrowhead). B: Angiography performed before embolization. C: Angiography performed after embolization; the arterial blood supply in the vicinity of the dental cyst with coil implantation (arrow) is blocked.

The lesion was diagnosed as a dentigerous cyst (differential diagnosis: an odontogenic tumor such as ameloblastoma). A biopsy was suggested to confirm the diagnosis preoperatively; however, as the patient wanted a one-stage operation and removal of the lesion, it was decided to perform a histopathological examination after total removal of the lesion. Therefore, the patient was scheduled for extraction of the third molar and removal of the cyst under general anesthesia. However, we considered the possibility of adhesions between the cyst and the branches of the maxillary artery and other arteries in the bone defect in the posterior wall of the maxillary sinus. Therefore, to ensure a safe surgical procedure, we scheduled the third molar extraction and cystectomy after vascular embolization. The target of embolization was the maxillary artery peripheral to the middle meningeal artery, and the direction of the artery was confirmed preoperatively using CT angiography (Figure 2A). A 4F catheter was placed in the right external carotid artery, angiography was performed, and a microcatheter was placed in the right maxillary artery under fluoroscopic guidance. A contrast agent was injected to confirm the presence of peripheral vessels in the maxillary artery (Figure 2B). A lidocaine solution was then injected at the distal end of the middle meningeal artery branch. After confirming the absence of abnormalities in the visual field or eye movements, the artery was embolized using three coils. The embolization procedure was completed after confirming the absence of the distal portion of the right maxillary artery (Figure 2C).

**Figure 3:**
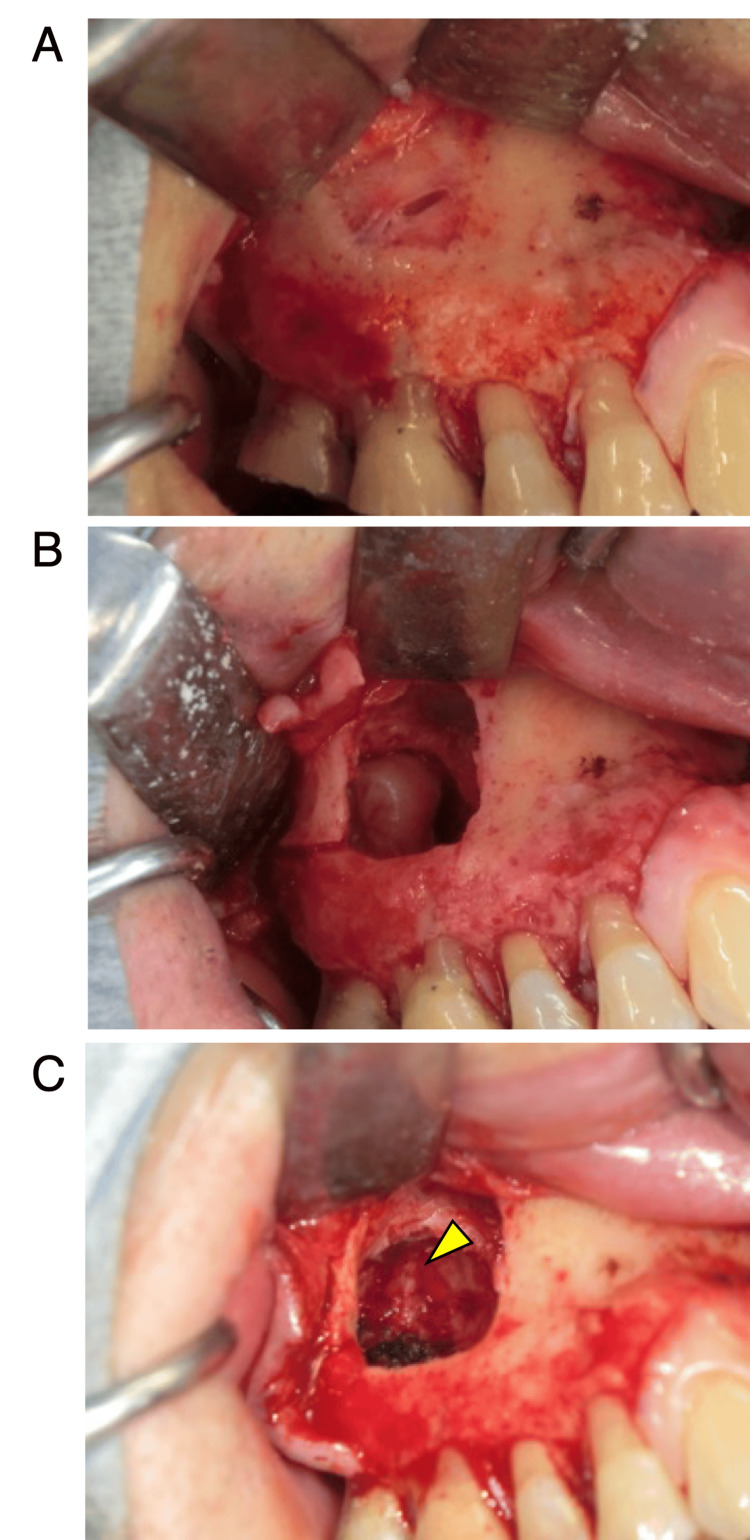
Intraoperative findings in cystectomy. A: Anterior wall of the maxillary sinus is thinned by the cyst and the exposed cyst wall. B: Exposed third molar after the sinus was opened using the Caldwell–Luc technique. C: Exposed descending palatine artery (arrowhead) after removal of the cyst.

The next day, the patient underwent a third molar extraction and a cystectomy under general anesthesia. Surgery was performed by ablating the anterior wall of the maxillary sinus using the Caldwell-Luc method (Figure 3A). A cyst was found in the maxillary sinus and the third molar, with the crown of the third molar inside the cyst (Figure 3B).

**Figure 4:**
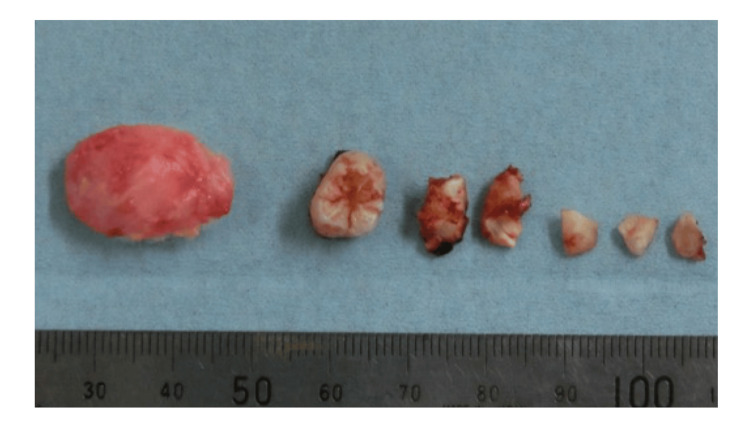
Exposed cyst after the extraction and the split-extracted third molar. Cyst capsule containing the crown of the tooth and the split-extracted third molar.

Because the third molar intersected the root of the second molar, the third molar was split and extracted. The cyst was attached to the thinning posterior wall of the maxillary sinus and was carefully dissected and extracted. After removal, the descending palatine artery was exposed in the maxillary sinus (Figure 3C); however, no arterial bleeding was observed due to the embolization performed the previous day. The excised cyst was round and smooth on the surface. The third molar, with a crown inside the cyst, was divided and extracted (Figure 4).

**Figure 5:**
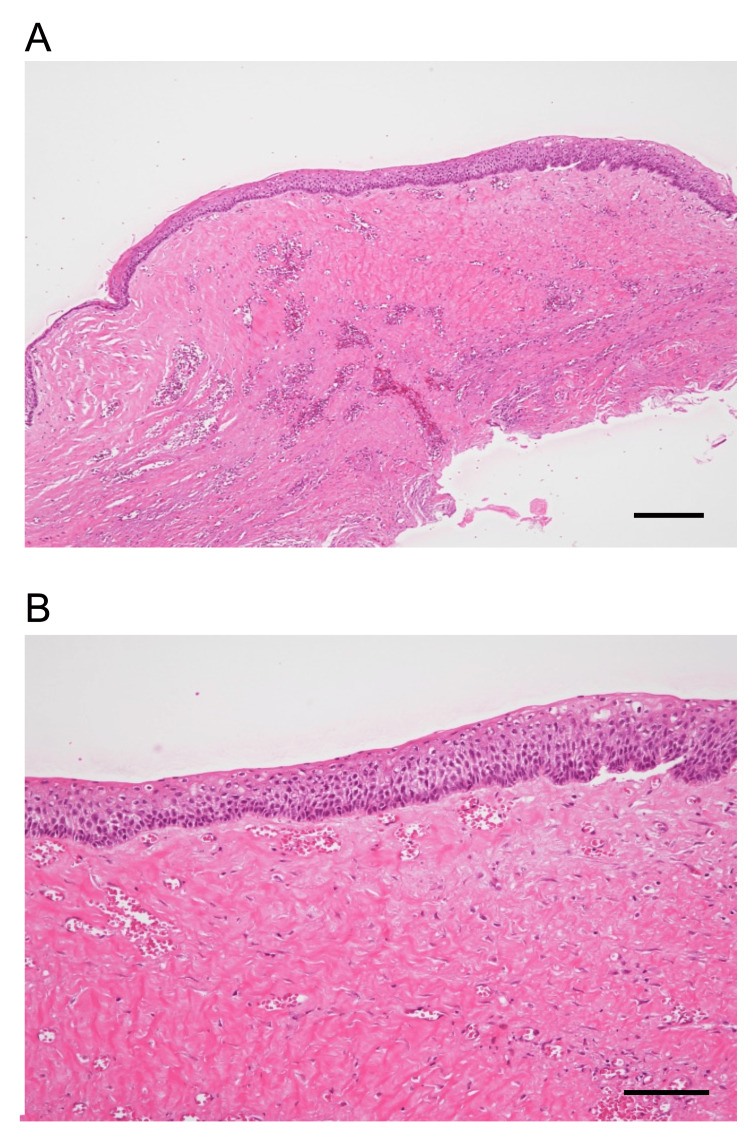
Histopathological specimen with hematoxylin and eosin staining. A cystic epithelium consisting of non-keratinized epithelium is present over the entire layer, with fibrous tissue growth (Scale bar: 200 $\mu$m). B: High magnification (Scale bar: 100 $\mu$m)

The final intraoperative blood loss was 120 mL (suction volume measurement). Histopathological examination of the whole excised specimen showed no tumor cells, such as in ameloblastoma, and a cystic epithelium composed of non-keratinizing epithelium with fibrous tissue growth (Figures 5A, 5B).

**Figure 6:**
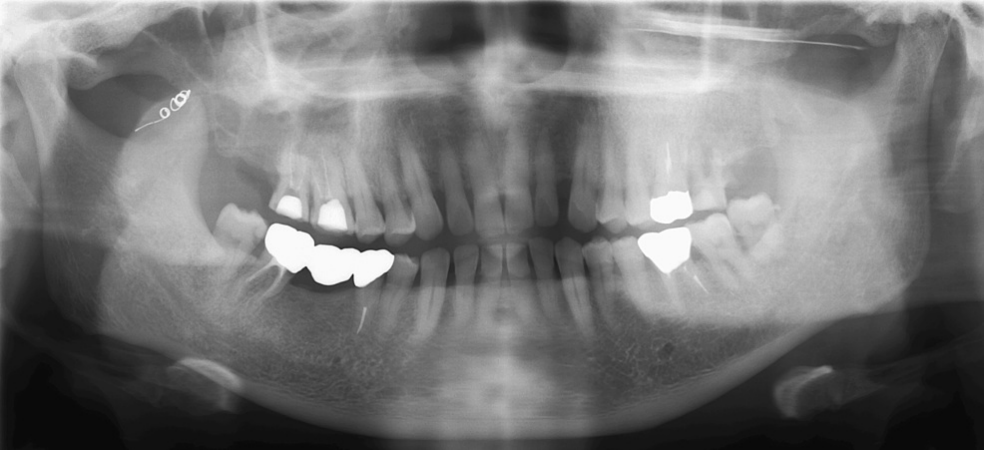
Postoperative panoramic radiographic image. The vascular embolization by the coil is maintained, and there is no evidence of cyst recurrence.

Hence, the pathologist made a definitive diagnosis of dentigerous cysts. No postoperative bleeding or complications associated with embolization were noted. The patient remained embolized with coils and remained in good health for four years after the procedure with no signs of recurrence (Figure 6).

## Discussion

The maxillary sinus is located directly above the maxilla; therefore, cystic diseases that occur in the maxilla often extend to the maxillary sinus. Surgical treatment for cysts extending to the posterior wall of the maxillary sinus may also involve the pterygopalatine fossa or pterygoid fossa, and the maxillary artery and its branches may coexist, making surgery difficult due to bleeding. In general, intraoperative bleeding is first treated with pressure hemostasis, ligation of blood vessels, and sutures. Particularly, ligation of the external carotid artery is considered effective when achieving hemostasis is difficult, and the procedure is performed occasionally [7,8]; however, it is invasive and can cause nerve paralysis. Moreover, there is a high risk of failure. Conversely, transcatheter arterial embolization can now be performed safely and accurately because CT angiography and magnetic resonance angiography have enabled detailed visualization of the vascular anatomy [9]. Endovascular embolization has been reported to be an effective hemostatic treatment for hemorrhage that is difficult to control locally in patients with cancer [10]. To reduce intraoperative bleeding in the present case, highly selective arterial embolization was performed preoperatively on the dominant artery at the resection site for a cystic lesion extending to the posterior part of the maxilla.

A coil was used for embolization. A catheter was inserted from the right external carotid artery into the right maxillary artery (i.e., the main artery of the maxilla), and a mixture of 2% lidocaine (1 mL) and normal saline (2 mL) was injected slowly, confirming no changes in the visual field or eye movements. Subsequently, the right maxillary artery was embolized distal to the bifurcation of the right middle meningeal artery using coils. A gelatin sponge is usually used as a temporary embolic material; however, because the gelatin that flows out of the microcatheter is carried by the bloodstream to the embolization site, there is a possibility of an unexpected inflow into the blood vessel, backflow, and embolization. Therefore, though expensive, a metal coil is desirable for achieving safe and reliable embolization. When preoperative prophylactic embolization is performed, the duration of embolization, resumption of circulation, and formation of peripheral collateral circulation are risk factors for the recurrence of bleeding [1]. However, in the present case, embolization was performed the day before the surgery; therefore, its impact was considered minimal as it was impossible for the embolized area to re-cross or form collateral channels within 24 hours before surgery.

Regarding the embolization site, embolization of the posterior superior alveolar artery, infraorbital artery, and descending palatine artery that branches from the pterygopalatine to the posterior maxilla of the maxillary artery was thought to reduce the amount of bleeding [11]. However, a branch of the middle meningeal artery may anastomose with the ophthalmic artery. Therefore, embolization at the origin of the middle meningeal artery should be avoided due to the risk of serious complications, such as decreased visual acuity due to retinal embolism [12,13]. No serious complications, such as ocular or facial abnormalities, were observed in this case. Pre-embolization is also recommended for diseases other than cystic lesions, such as maxillary carcinomas or drug-induced osteonecrosis of the jaw, when the resection site is close to the maxillary artery. However, a limitation of this technique is the position of the catheter, as the diameter of vessels in the peripheral branches of the maxillary artery (e.g., the posterior alveolar artery) is smaller than that of the middle meningeal artery. In addition, the vessel must be healthy to perform endovascular embolization. It cannot be performed in patients with plaque formation due to atherosclerosis or with diabetes mellitus, which causes fragile vessels that are prone to collapse. Complications of preoperative arterial embolization include paresthesia around the affected area, fever, impaired mouth opening, and facial nerve palsy [14]. In serious cases, there is loss of vision due to embolization of the ophthalmic artery and reflux of the embolus into the internal carotid artery [15]. Therefore, it is important to confirm the surgical technique, extent of resection, and vascular running and to discuss the embolization site with a radiologist before the operation.

## Conclusions

Preoperative embolization with coils is extremely useful for managing cystic lesions beyond the posterior wall of the maxillary sinus to avoid hemorrhage from the maxillary artery branches. Moreover, peripheral embolization from the middle meningeal artery can minimize complications from embolization.

Thus, it is important to thoroughly study the location of the lesion and vessels with CT beforehand because avoiding the risk of preoperative bleeding can reduce the amount of blood loss and enable a safer procedure.
